# The Impact of Epidural Analgesia on Immobility and extended Hospital Stay After Periacetabular Osteotomy

**DOI:** 10.1007/s00402-024-05331-4

**Published:** 2024-05-07

**Authors:** Sufian S. Ahmad, Nils Becker, Laura-Vanessa Grap, Henning Windhagen, Marco Haertlé

**Affiliations:** Department of Orthopaedic surgery, Hannover Medical School, Anna-von-Borriesstr. 1-7, 30625 Hannover, Germany

**Keywords:** Periacetabular osteotomy, Epidural analgesia, Pelvic osteotomy, Hip dysplasia

## Abstract

**Aims:**

Early mobilisation after periacetabular osteotomy (PAO) represents an important goal after surgery. The purpose of this study was to determine whether the use of epidural aznalgesia (EA) is associated with prolonged immobility and an increased length of stay (LOS) after PAO surgery.

**Methods:**

From January 2022 to July 2023, the study included a cohort of 150 PAO procedures all performed by the same surgeon (SSA). Patients were categorized into two distinct groups: those who received epidural analgesia (EA) (79 PAOs) and those who did not receive EA (71 PAOs). "Ready for discharge" was defined as the ability to ascend and descend a standardized flight of stairs independently. Multivariable linear regression was used to identify additional factors influencing LOS after PAO.

**Results:**

Patients in the EA group were ready for discharge 5.95 ± 2.09 days after surgery which was significantly longer than in the No EA group´s average of 4.18 days ± 2.5, (*p* < 0.001). While the reduction in the number of patients experiencing pulmonary embolism in the No EA group did not reach statistical significance, it still demonstrated a relevant decrease from two patients within the EA group (2.53%) to 0 (0%) in the No EA group. The active engagement of the surgeon in mobilising patients led to a substantial reduction in LOS, decreasing it from 5.81 ± 2.18 days to 2.2 ± 0.77 days (*p* < 0.001). Multivariable analysis revealed five independent factors influencing the LOS following PAO which included absence of EA, surgeon-led mobilisation within 24 h after surgery, postoperative hemoglobin levels, BMI, and prior experience with PAO surgery on the contralateral side.

**Conclusions:**

Opting against the use of EA in patients undergoing PAO is advisable, as it will result in extended postoperative immobility and the associated risks. Additionally, the active participation of the surgeon in the mobilisation process is strongly recommended.

**Supplementary Information:**

The online version contains supplementary material available at 10.1007/s00402-024-05331-4.

## Introduction

Periacetabular osteotomy (PAO) has gained wide recognition as a powerful and effective procedure for the treatment of abnormal morphologies of the acetabulum since its first description in 1988 [1–3]. The primary advantage of PAO over other osteotomy techniques was underlined by maintanance of the integrity of the posterior column. The inherent stability of an osteotomy that maintains continuity of the hemipelvis was thought to allow earlier mobilisation and recovery [4, 5]. Therefore, the goal of early moblisation after PAO surgery deserves more attention in the current era of fast-track orthopedic surgery [6]. The postoperative in-hospital recovery in terms of independent mobilisation and the length of stay (LOS) has not yet received sufficient attention in the scientific literature related to PAO. This may be due to the complexity and the technical demands of the procedure, which have traditionally overshadowed the implementation of rapid recovery concepts [7, 8]. There is a notable trend towards less invasive PAO surgery and the range of indications are expanding to include retroversion and milder cases of hip instability, emphasizing the need for an approach to reduce the recovery period after this procedure [9–12]. Rapid recovery concepts that have gained sigificant recognition in arthroplasty surgery will inevetibaly be transferred to complex osteotomies. This aspect holds significant relevance in optimizing perioperative care and calls for dedicated scientific research.

The aim of the study was to determine the influence of perioperative epidural analgesia (EA) on independent mobilisation after PAO surgery. The secondary aim was to identify further potential factors influencing independent mobilisation.

## Methods

### Patients

**Fig. 1:**
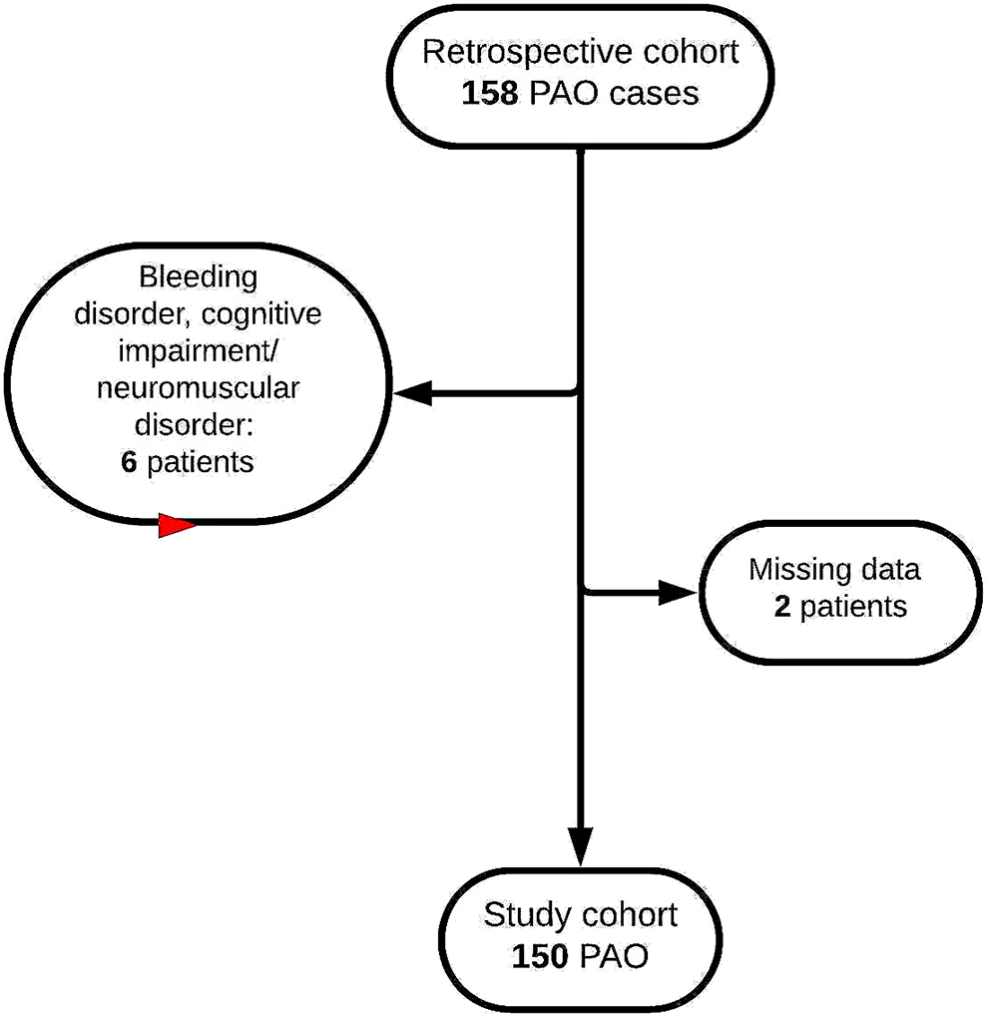
Flowchart illustrating the process of patient inclusion

A consecutive series of 158 PAO procedures performed between January 2022 and July 2023 by a single surgeon were considered for inclusion in this study. All patients signed a written consent and ethical approval was received for the conduction of the study (10405_BO_K/11334_BO_K). Patients with syndromic disease, cognitive disability, bleeding disorder requiring substitution therapy, or incomplete data, were excluded (Fig. 1). Table I shows demographic patient characteristics.

### Description of surgery

The surgery was performed as previously described [13, 14]. The approach involved preservation of the rectus tendon and no stripping of the iliocapsularis muscle from the capsule. An incomplete ischial cut was performed with fluoroscopic control and ensuring notching of the entire cortical width mediolaterally. Next, the pubic osteotomy is performed medial to the pubic emenentia using a straight osteotome in an anterolateral to posteromedial direction. The supracetabular osteotomy was performed starting at the lower border of the anterior superior iliac spine aiming towards the floor and ending 1 cm lateral to the pelvic brim. The retroacetabular cut was aimed toward the ischium and fluoroscopic assistance was applied to ensure a perfect trajectory. Completion of the osteotomy was achieved by freeing the fragment and disrupting the posterior inferior hinge from the inside of the pelvis with a curved osteotome. A Schanz pin was used for re-orientation and fixation was achieved with 2–3 4.5 mm large fragment screws. Autologous cell salvage was used intraoperatively.

Patients were allowed to partially weight bear with 15 kg for 4 weeks immediately after surgery. A follow-up X-ray was then taken after four weeks, prior to weight bearing.

### Variables and endpoints

LOS was defined as the primary outcome measure. In order to be considered ready for discharge, each patient had to be able to ascend and descend a standardized flight of stairs, independently.

The standardized set of stairs consisted of 12 steps with a tread width of 32 cm and a tread height of 15 cm. Patients used elbow crutches to assist in mobilisation. The goal of independently ascending and descending all steps had to be accomplished prior to discharge. The timepoint of achieving this goal was documented by the treating physician or physiotherapist. ΔCRP increase, Δhemoglobin reduction as well as average pain at rest and during movement were noted. Pain was documented using a visual analog scale (VAS), with a value of 0 referring to no pain and 10 to maximum pain. Blood tests were performed on postoperative days one, three, and five. Pain assessment was conducted thrice daily.

Stratification into two groups was performed based on the presence or abscence of EA. The reason why patients did not receive EA was based on an interdisciplinary decision to discontinue the use of EA for pain management in PAO surgery.

A power analysis was conducted based on the primary hypothesis that absence of EA would reduce hospital stay. Power anaylsis demonstrated that a minimum of 25 patients per group would be required to achieve a statistical power of 80%, given a significance level (alpha) of 0.05. The following input variables were considered for multivariant regression analysis: 1) age in years, 2) Surgical duration, 3) Type of non-steroidal anti-inflammatory drugs (NSAIDs) administered noted as a dichotomous variable, with two options either 3 × 600 mg oral Ibuprofen or 2 × 40 mg intravenous parecoxib, 4) Involvement of the surgeon in patient mobilisation defined as the physical presence of the surgeon during initial mobilisation out of the bed in order to provide assurance and facilitate the first steps with crutches, this was noted as a ‘yes’ or ‘no’ answer and was independent of the standard physiotherapy care that patients received once per day. 5) Previous experience with PAO on the contralateral side was noted as a dichotomous variable indicating whether the patients had previously undergone a PAO procedure. 6) Gender as a dichotomous variable. 7) The use of EA. 9) Height (cm), weight (KG) and BMI as a continuous variable. 10) The lowest haemoglobin level during the hospital stay after surgery.

Normally distributed data were presented as mean (± standard deviation). Comparison between means was performed using analysis of variance (ANOVA). A multivariate linear regression model was used to determine factors influencing LOS. The 10 above mentioned input variables were considered. Reverse adjustment was then performed by systematically excluding the least significant factors one at a time. A p-value of < 0.05 was considered statistically significant. SPSS version 29 (IBM statistics, Armonk, NY, USA). Two patients were excluded from the analysis due to missing data.

## Results

**Fig. 2:**
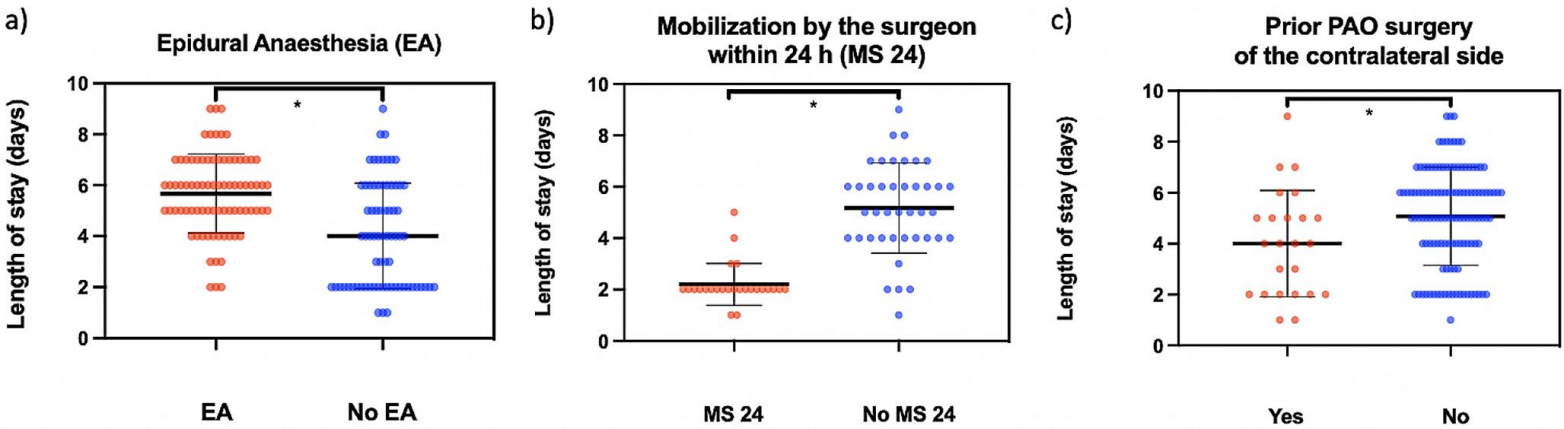
**A** Plot demonstrating that the absence of epidural anesthesia (EA) significantly reduced the length of stay after PAO (4.18 ± 2.5 days vs. 5.95 ± 2.09 days) after PAO. (EA *n* = 79 vs. no EA *n* = 71; * p = < 0.001) **B** Plot demonstrating that mobilization by the surgeon within 24 h after periacetabular significantly reduced length of stay (2.2 ± 0.82 nights vs. 5.39 ± 2.42 nights) (MS 24 *n* = 28 vs. no MS 24 *n* = 43; * *p* = 0.001) **C** Plot showing that prior PAO surgery of the contralateral side significantly shortens the length of hospital stay after PAO (4.00 ± 2.09 days vs. 5.35 ± 2.46 days; *p* = 0.006) (yes *n* = 24 vs. no *n* = 126; *p = < 0.01, *p = < 0.001)

The study included 150 hips of 126 patients who underwent PAO. Among these, 79 patients (52.7%) received EA for perioperative pain management (Table 1). Patients who had received EA required two additional days to successfully achieve the goal of independent stair climbing with crutches, as illustrated in Fig. 2A. The use of EA was also identified as an independent factor for prolonging the LOS after PAO in the adjusted regression model (Table 2). In the subgroup of patients who did not receive EA (71 patients), a total of 29 patients (40.85%) were mobilized by the surgeon within 24 h after PAO. As a result, there was a significant reduction of LOS by 3.2 days (Fig. 2B). Multivariable linear regression confirmed surgeon-led mobilisation as a factor reducing LOS (Table 2).

**Table 1:** Descriptive data epidural anesthesia (EA) vs. absence of epidural (no EA) anesthesia

Number of patients	EA	No EA	*p*-value
Additional interventions	79/150 (52.66%)	71/150 (47.33%)	
Femoral neck osteoplasty	21/79 (26.58%)	15/71 (21.13%)	0.4347
Rotational femur osteotomy	2/79 (2.53%)	3/71(4.23%)	0.564
Hardware removal	2/79 (2.53%)	3/71(4.23%)	0.564
Ø Age (years)			
Sex	29.53 ± 9.96	29.04 ± 7.12	0.3923
Male	25/79 (31.65%) 54/79 (68.35%)	17/71 (23.94%) 54/71 (76.06%)	0.2942
Body mass index (kg/m^2^)			
Clinical data	26.52 ± 16.1	24.81 ± 4.87	0.1945
Surgical duration (min)	76.63 ± 20.92	74.17 ± 31.07	0.2832
Length of stay (days)	5.95 ± 2.09	4.18 ± 2.5	< 0.001
ΔHaemoglobin (g/dl)	4.34 ± 1.5	4.37 ± 2.33	0.4733
ΔCRP (mg/dl)	4.67 ± 3.1	4.49 ± 3.98	0.3777
VAS at rest (1–10)	2.17 ± 0.7	2.58 ± 0.93	0.0013
VAS on movement (1–10)	3.27 ± 0.92	3.51 ± 1.19	0.0929
Radiographic data			
Preoperative LCEA (°)	17.07 ± 6.02	17.29 ± 7.9	0.4252
Postoperative LCEA (°)	30.3 ± 4.68	31.51 ± 6.46	0.087
Preoperative acetabular index (°)	11.83 ± 6.32	11.81 ± 686	0.491
Preoperative anterior wall index	0.44 ± 0.23	0.48 ± 0.19	0.139
Preopertative posterior wall index	0.67 ± 0.26	0.75 ± 0.24	0.021
Preoperative alpha angle (°)	56.08 ± 15.0	56.38 ± 15.26	0.4536
Pulmonary embolism			
	2/79 (2.53%)	0/71 (0.00%)	0.1547

**Table 2:** Multivariable linear regression analysis for independent factors influencing length of hospital stay (LOS) after PAO

Dependent factor	Independent factor	Confidence interval				
LOS (days)		Beta (β)	Regression coefficient (B)	Lower Limit	Upper Limit	Sig.
	Mobilisation by the surgeon within 24 h after PAO	-0.303	-3.343	-5.127	-1.513	< 0.001
	Prior PAO surgery of the contralateral side	-0.204	-1.294	-2.344	-0.243	0.016
	Lowest postoperative hemoglobin level	-0.246	-0.351	-0.586	-0.115	0.004
	BMI	-0.303	0.095	0.011	0.179	0.027

**Fig. 3:**
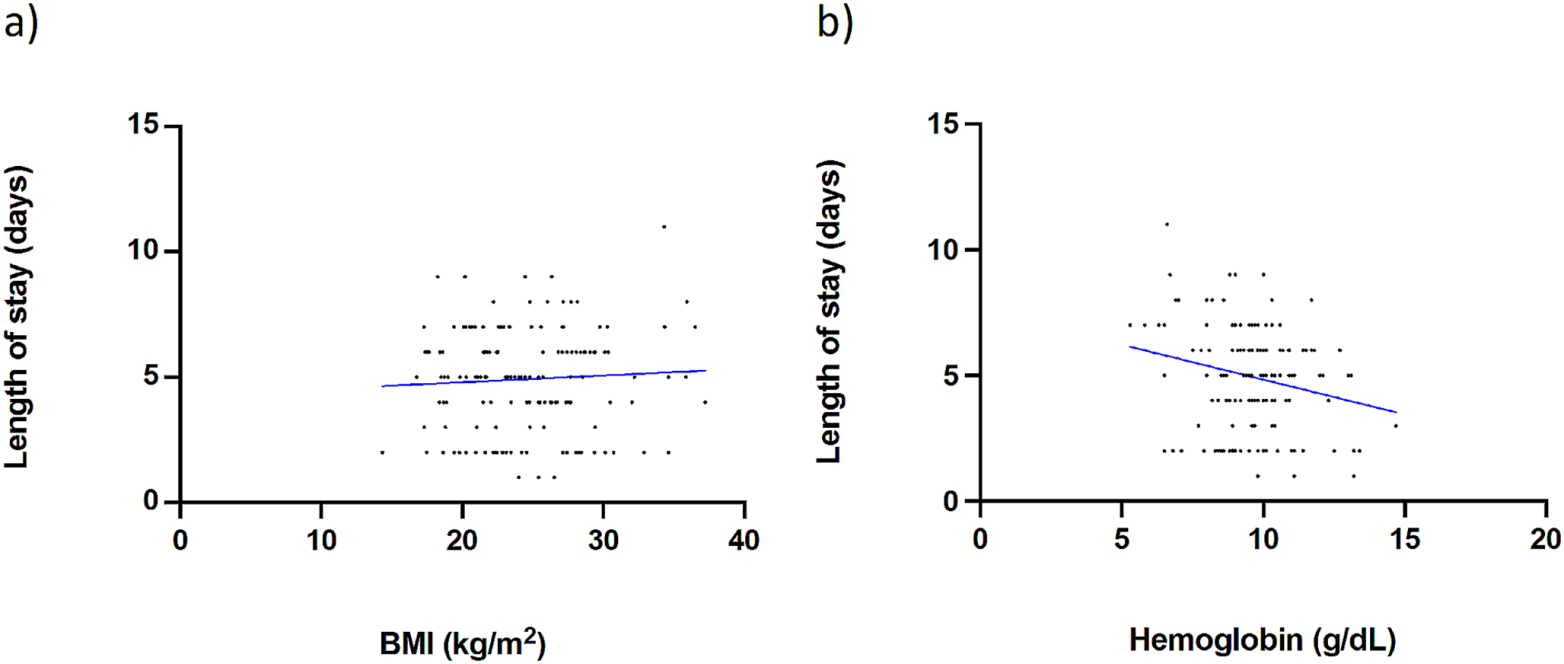
**A** Scatter plot showing a significant correlation between BMI and timepoint of discharge after surgery (*p* = 0.027) **B** Scatter plot illustrating the significant association between hemoglobin level (g/dL) and length of stay *after PAO* (*p = 0.004*)

Multivariate linear regression determined further factors influencing the LOS after PAO surgery (Table 2). These included BMI, where increased BMI was associated with delayed discharge after PAO (Fig. 3A). Additionally, prior experience with contralateral PAO surgery was associated with enhanced postoperative mobility and an earlier state of readiness for discharge following the subsequent PAO procedure (Table 2; Fig. 2C). It was also observed that the lowest postoperative hemoglobin levels showed a direct influence on the LOS, as shown in both univariate and multivariate regression (Table 2; Fig. 3B).

Although the incidence of pulmonary embolism (PE) was higher in the group receiving epidural analgesia (EA), the difference was not statistically significant (p = 0.1547). Within the EA group, two cases (2.53%) of PE were noted in female patients using contraceptive medication, with one occurring during the hospital stay and another three weeks later. On the other hand, PE events were not recorded in the group that did not receive EA (Table 1).

## Discussion

The study revealed a significant reduction in LOS by two days among patients who had not received an epidural catheter. The second finding was associated with the surgeon’s engagement in the initial patient mobilisation process, resulting in a further average reduction of 2 days.

Extensive evidence from the literature highlights substantial benefits of early mobilisation in patients with pelvic fractures, to the point where it is considered a fundamental component of the resuscitation process [15]. Early mobilisation is particularly valued for its effectiveness in reducing the risk of thromboembolic events.

The development of thrombosis is a multifactorial process that occurs in the early postoperative period and represents a serious complication with reported mortality rates of up to 10 to 15%. In addition to prolonged immobilisation, other risk factors contributing to the development of thrombosis and pulmonary artery embolism after pelvic surgery include the duration of surgery, gender, medication, blood clotting disorders and obesity [16, 17]. In addition to the consistent implementation of pharmacological thromboembolic prophylaxis and individual risk stratification of each patient, early mobilisation in particular represents an effective measure to reduce the rate of thrombosis and pulmonary embolism after surgery [18]. Hence, prolonged immobility after PAO due to EA represents a modifiable risk factor that can potentially compromise patient safety.

Furthermore, it is important to note that the dysplastic population primarily comprises females of child-bearing age, with a likelihood of taking birth control pills, inherently at a higher risk of developing a thromboembolic event [19]. Therefore, early mobilisation is of major clinical significance in patients undergoing PAO surgery, underlining the clinical relevance of the research question of this study. Although the data on preventing PE in the absence of EA lacked statistical significance, a clear pattern emerged indicating a potential beneficial impact of increased activity resulting from the absence of EA in reducing the overall incidence of PE following PAO from 2.5 to 0% in this study group. The possible negative impact of EA on the occurrence of PE have been already described in the context of abdominal surgery [20].

There is a lack of research comparing the use and non-use of an epidural catheter for perioperative pain management in PAO surgery. However, a recent study highlighted the advantages of early removal of the epidural catheter following PAO by demonstrating a shorter LOS and reduced opioid consumption [21]. Furthermore, a review by Millis et al. mentioned that epidural was abandoned in the authors’ institution [22]. Hence, the prevailing trend in the literature appears to lean against recommending the use of epidural analgesia in PAO surgery.

Another finding pertained to the influence of the surgeon’s presence during the initial mobilisation process after surgery. It seems that the assurance that is provided by the initial presence of the surgeon enables accelerated progress during the entire hospital stay. The third finding demonstrated a relationship between BMI and LOS after PAO. This association could be attributed to the initial challenges associated with mobilizing a larger body mass and the increased requirement for upper extremity control when managing crutches. Several studies in the literature have examined BMI, and some have indicated a higher risk of perioperative complications following PAO in patients with a BMI greater than 30 kg/m², categorizing this patient group as high risk [10, 23, 24]. It is therefore advisable to explicitly discuss potential postoperative mobility challenges related to BMI prior to surgery and emphasize the importance of more intensive postoperative physiotherapy in order to mitigate BMI-related immobility and the associated risks.

**Fig. 4:**
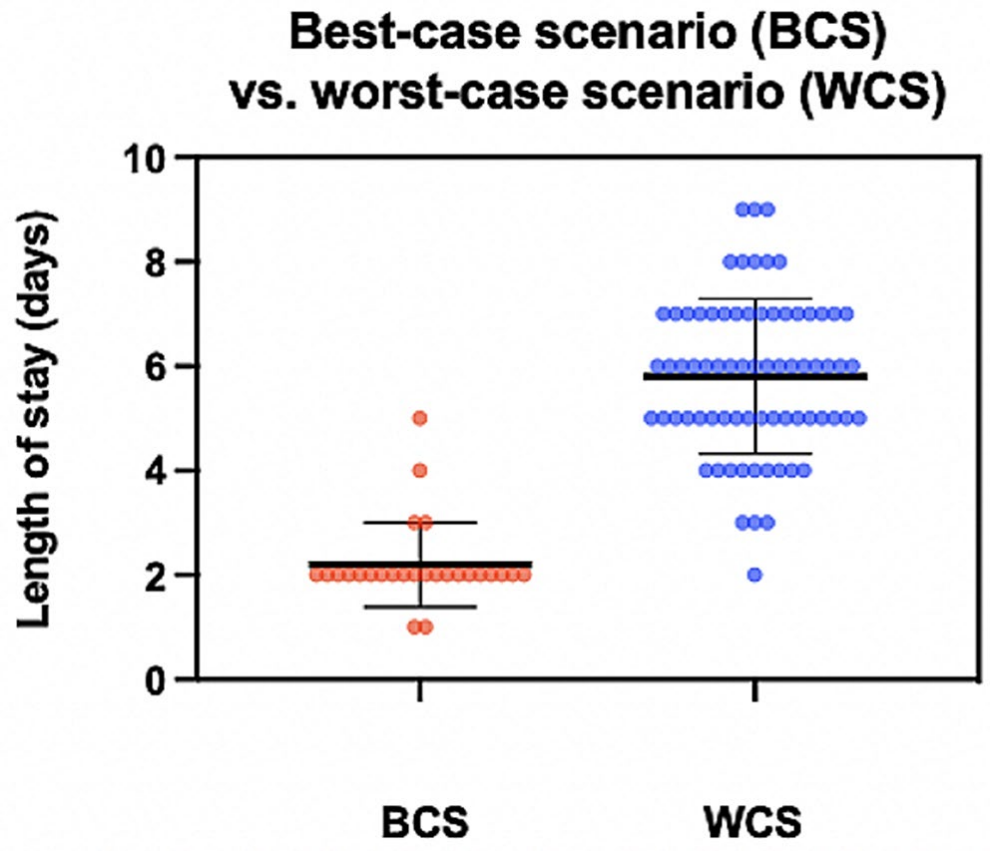
The best-case scenario was defined as the combination of modifiable factors, including the absence of epidural anesthesia and mobilization by the surgeon within 24 h after surgery. Conversely, the worst-case scenario was characterized by the absence of both modifiable factors. The group experiencing the best-case scenario showed a significant reduction in LOS after PAO, compared to the worst-case scenario (2.19 ± 0.8 vs. 6.09 ± 2.06 days) (BCS *n* = 25 vs. WCS *n* = 75; * *p* = 0.001)

Another significant result indicated that prior experience with PAO surgery on the contralateral side was linked to a reduction of LOS after surgery. This is proof of the fact that there are potentially modifiable factors that influence early recovery. This includes experience with the use of crutches, less fear, and more trust in the stability of the construct. Hemoglobin levels after surgery were also shown to determine postoperative discharge. This has been previously discussed in studies looking into fast-track hip and knee replacement [25]. Therefore, minimizing blood loss during surgery should be considered an important surgical goal. There is no doubt that a pelvic osteotomy is associated with increased blood loss. However, blood management concepts including maintenance of a low mean arterial pressure, autologous cell salvage, and using bone wax to reduce bleeding from the nutritional foramen are potential intraoperative actions that could contribute to controlling blood loss. Considering the modifiable factors influencing early hospital discharge, the best-case scenario (BCS) involves the combination of two factors: mobilisation by the surgeon and avoiding EA, the combination of which results in a significantly reduced LOS after PAO by an average of 3.8 days. (Fig. 4)

This study has several limitations that should be considered. Firstly, the variable of mobilisation defined by the surgeon was applied randomly and based on the treating surgeon’s schedule, rather than being performed in a controlled manner. However, it is important to note that there was no bias regarding patient selection. Additionally, statistical adjustments were made to account for potential confounding variables. Another limitation is that hospital discharge was slightly influenced by social factors, such as family and personal arrangements, which occasionally led to a one-day delay in discharge for a minority of patients. To address this issue, the time period necessary for patients to successfully climb a standardized set of stairs served as a benchmark for determining readiness for discharge. Patients were granted discharge as soon as they successfully achieved this goal from the medical side.

## Conclusions

Prompt mobilisation following PAO surgery stands as one of the initial and paramount postoperative objectives. This study has unveiled various factors, both modifiable and non-modifiable, that influence early mobilisation, ultimately resulting in a shorter hospital stay. These factors encompass early surgeon-led mobilisation, the absence of an epidural catheter, BMI, postoperative hemoglobin levels, and prior experience with PAO on the contralateral side.

The findings of the study advise against the use of an epidural catheter in PAO patients, as it would lead to extended postoperative immobilisation, with its attendant risks. Furthermore, active surgeon involvement in mobilisation is strongly encouraged, given its substantial positive impact on early recovery.

### Electronic supplementary material

Below is the link to the electronic supplementary material.

Supplementary Material 1

Supplementary Material 2

Supplementary Material 3

Supplementary Material 4

Supplementary Material 5
